# Mr. Earnest's Case of Lepra

**Published:** 1807-04

**Authors:** Robert Earnest

**Affiliations:** House Surgeon to the Sheffield General Infirmary


### *To the Editors of the Medical and Physical Journal.*

GENTLEMEN,

UPON looking over the numerous remedies that are on record, but more especially those which are enumerated by Doctor Willan, for the cure of that loathsome disease, called Lepra; I am sorry to find that very few indeed are in any estimation for their efficacy in the removal of this cutaneous disorder, which has, in many instances, baffled the most skilful practitioner, by its resisting the action, and the well-known good effects, of many of the most powerful medicines. This disease also, I fear, like some others to which the human body is incident, has not been improperly placed under the appellation of the opprobrium medicorum. It is therefore the office and duty of every practitioner, to endeavour as much as is within the reach of his power, to diminish the number of incurable maladies; and this can only be done by his communicating to the public the result of his own experience, in attempting to cure obstinate and baneful diseases.

It is from this motive I send you the enclosed; though only a solitary case of inveterate lepra vulgaris, which was successfully treated by the kali sulphuratum, it is deserving of attention. If upon a perusal of the case, you should deem it worthy a place in your very useful publication, you will oblige me by inserting it in the next Number.

I am, &c.

ROBERT EARNEST,

*Feb.* 10, 1807. House Surgeon to the Sheffield General Infirmary.

### CASE.

The subject of this case is a girl, 15 years of age; and although so young, she had been in service two years; she is very stout of her age, her complexion is dark, her skin coarse, and her hair of a dark brown colour. She has menstruated regularly for the last two years; and excepting being troubled with copious perspirations from the soles of the feet, (which may be considered more as a salutary secretion than otherwise) she has uniformly enjoyed good health.

During the menstruating period, which happened some time in the month of May, 1805, after washing the kitchens and other offices, she unguardedly at this critical time, walked about the damp floors without shoes; in consequence of which, the flow of the catamenia was checked immediately, and she was seized with the following symptoms. Nausea at the stomach, head-ache, heats and rigors by turns, loss of appetite, costiveness, and debility. From this time the patient continued to be much indisposed with febrile complaints, for nearly a month; but at length she was relieved by Nature throwing to the surface of the body, the disease called by Nosologists, Lepra. This disease appeared first upon the integuments, covering the patella of the left knee, and next about the olecranon of the left arm. The lepra afterwards attacked such particular parts progressively, until it became almost universal, pretty much in the same manner Dr. Willan mentions, when treating of this disease. See Willan, on Cutaneous Diseases, Ord. ii. page 112 et seq. The disease in this case was by far the most malignant in the following parts. 1. The hairy scalp was one continued dry incrustaceous mass, irregularly elevated into small eminences one above another, which were truly analogous to a cauliflower; and was much the longest in getting well. 2. The skin of the fore-arms from the elbow to the first joints of the fingers, had also an incrustaceous covering; but of a moist nature, there being a profuse exudation of very ichorous matter, forming furrows in different parts of the skin, wherever it had an outlet. 3. The integuments surrounding the knee joints,* and parts adjacent, for a considerable distance, both above and below, were affected in a manner similar to the arms.

There were some patches upon the forehead, temples, cheeks, and chin.

When the patient walked, or used her arms, the skin about the joints was often lacerated, which produced more or less hæmorrhage, and subsequent pain and stiffness in the parts affected.

Four months from the first appearance of the complaint had elapsed before the patient applied at the Infirmary for advice. The state of the disease at this time, (September) was as I have before described; but the girl's general health had become better, as she could eat, drink, and sleep tolerably well; and she again discharged the menses at regular periods.

The

---

* On comparing this patient's knees with plate viii. ord. ii. at page 212, in Dr. Willan's work upon Cutaneous Diseases, which is intended to represent Lepra upon these parts; I found that the plate was really a fac simile of the disease. As are also those representing the same disease upon the arms.

The remedies first employed were as follow. On the twenty-first of September she took an emetic draught, and in the morning following, she took a purging bolus with calomel and rhubarb. On the twenty-third, she began with a course of Dr. Plummer's pills, and the decoction of the woods, accompanied by a good diet. The tepid bath was also used twice a week. These remedies were continued for nearly a month, without any apparent benefit, and on account of the patient having at this time feverish symptoms, such as head-ache, nausea at the stomach, and a greater sense of heat upon the skin generally, the above medicines were left off altogether: and now the emetic draught and the purging bolus were again repeated.

The day following these evacuants, the patient was put upon the nitrum purificatum twice a-day. She began with half a scruple, and the dose was gradually augmented by five grains at a time daily, until she was able to take five scruples at a dose bis die. This medicine was persevered in for three weeks, and the only good effect it had, was by acting as a refrigerant; but it neither altered the state of the pulse, nor the appearance of the disease upon the skin. The nitre was now omitted, and the patient began with the kali sulphuratum, compounded as follows. ℞. Kali sulphurat. ʒij. ad ʒvj.* Solv. in aq. pur lib. ii. fiat solutio cap. unc. iv. bis in dies.

℞. Kali sulphurat. ʒj. ad ʒiv. solv. in aq. pur. lib. ii. fiat lotio bis die utend.

Illin. capiti. ung. sulphur. mane et vespere.

Ut. balneo tepid. c. kali sulphurat. ℥ii. bis in septimana.

In regard to the modus operandi of these remedies, I shall only observe, that the solution operated as a gentle aperient once or twice a-day. The lotion from the smarting it produced, seemed slightly caustic, and acted as a discutient. The ointment proved highly serviceable to the head, as it loosened and separated the disease gradually, and left the scalp sound.

The sulphurated tepid bath acted as a relaxant to the

 skin,

---

* In dissolving the kali sulph. in water, the sulphur is speedily precipitated, still the liquid to the smell and taste seems to be strongly impregnated with it, and by shaking the bottle well every time it is used, very little of the sulphur is wasted.

Neither this patient nor others, who have taken this medicine, have ever complained of having experienced any unpleasant effects from it, either in the stomach or the bowels.

skin, and greatly relieved the tension and stiffness of the extremities, of which the patient often complained.

When the above remedies had been persisted in for nearly a month, there were evident signs of general amendment, consequently they were ordered to be continued; and at length the disease began gradually to go off, in the manner described by Dr. Willan.*

These last indications were begun on the twenty-sixth of October, 1805, and was persevered in until the twenty-first of March, 1806, when the patient was discharged perfectly well.

### *Further Remarks upon the Case.*

The girl remained quite free from the smallest appearance of the malady, from the twenty-first of March until the twelfth of September. At this period, the disease made its appearance again about the elbows and knees; but in a much milder form, and without any evident cause, or constitutional ailments. The lepra now came out in the form of dry, white, lamellated scaly patches, unaccompanied by any inflammation of the contiguous parts.

Although the kali sulphuratum was before the specific remedy, it was thought right at this time to give the hydrargyrus muriatus, along with the decoctum lignorum, a fair trial. Accordingly the patient began with very small doses in the form of pills, which were gradually augmented, until the gums became slightly tender. And the parts affected, were anointed morning and evening with the following liniment. ℞. Sapon mollis ℥iss. Kali sulphurat. pulv. piper. nig. an. ʒiij. Ut fiat liniment. bis die utend.†

These remedies were persisted in six weeks without any intermission, and without any apparent advantage; therefore they were left off, and recourse again was had to the kali sulphuratum. This medicine was exhibited in the way I have before described, and which was continued seven weeks, at the end of which time there was not the smallest vestige of the disease to be seen, and the girl was a second time discharged cured.

To

* See Willan on Cutaneous Diseases, ord. ii. page 116.

† This liniment is prepared as follows. The kali sulph. is first rubbed down, which becomes more or less fluid, then the pepper in fine powder is added, and lastly the soap; the whole are then intimately mixed together in a mortar. This liniment has been of essential service in several cases of Tinea Capitis.

